# Microbial nitrification and acidification of lacustrine sediments deduced from the nature of a sedimentary kaolin deposit in central Japan

**DOI:** 10.1038/s41598-021-81627-4

**Published:** 2021-02-10

**Authors:** Tetsuichi Takagi, Ki-Cheol Shin, Mayumi Jige, Mihoko Hoshino, Katsuhiro Tsukimura

**Affiliations:** 1 Research Institute for Geo-Resources and Environment, Geological Survey of Japan, National Institute of Advanced Industrial Science and Technology (AIST), Tsukuba, Japan; 2 RIHN Center, Research Institute for Humanity and Nature, Kyoto, Japan; 3 Faculty of Education, Osaka-Ohtani University, Tondabayashi, Japan

**Keywords:** Biogeochemistry, Environmental sciences, Solid Earth sciences

## Abstract

Kaolin deposits in the Seto-Tono district, central Japan, were formed by intense kaolinization of lacustrine arkose sediments deposited in small and shallow inland lakes in the late Miocene. Based on mineralogical and stable isotopic (Fe, C, N) studies of Motoyama kaolin deposit in the Seto area, we concluded that it was formed by microbial nitrification and acidification of lacustrine sediments underneath an inland lake. Small amounts of Fe–Ti oxides and Fe-hydroxide in the kaolin clay indicated that iron was oxidized and leached during the kaolinization. The field occurrences indicate that leached ferric iron precipitated on the bottom of the kaolin deposit as limonite crusts, and their significantly fractionated Fe isotope compositions suggest the involvement of microbial activity. The C/N ratios of most of the kaolin clay are distinctly higher than those of modern lacustrine sediment. Although, the possibility of a low-temperature hydrothermal origin of the kaolin deposit cannot be completely ruled out, it is more likely that acidification by dilute nitric acid formed from plant-derived ammonia could have caused the kaolinization, Fe oxidation and leaching. The nitrate-dependent microbial Fe oxidation is consistent with dilute nitric acid being the predominant oxidant.

## Introduction

Kaolin (sensu lato) is one of the commonest minerals on the Earth’s surface. It is the final weathering product of crustal rocks in temperate regions. Understanding its genesis is a key to solving not only terrestrial weathering processes but also global material recycling. Kaolin occasionally forms mineral deposits ranging from a few hundred to several hundred meters in length, and has long been mined for porcelains/potteries, refractories and paper filler^1^. Commercially important kaolin deposits have been genetically divided into hydrothermal, sedimentary and metamorphic types^2,3^. Sedimentary deposits, the most common type, can be subdivided into detrital, residual and pedogenic/diagenetic but, in practice, the processes overlap. The process of chemical leaching/bleaching in sedimentary kaolin deposits in environments that are thought to be neutral or alkaline has not been fully elucidated. One possible process is mineral degradation by acid solutions produced by microbial activity. The importance of this has been highlighted by studies of kaolin deposits in coastal sediments in the Southeastern United States^4,5^. However, few studies on microbial involvement have been reported because possible microbial reaction pathways are numerous, and their traces are unlikely to remain in kaolin deposits.

In this study, we report a newly proposed origin of a kaolin deposit in Miocene lacustrine sediments in central Japan. The deposit was probably formed by intense chemical leaching and kaolinization caused by microbial nitrification and acidification of lacustrine sediments underneath a shallow inland lake.

### Geological background

**Figure 1:**
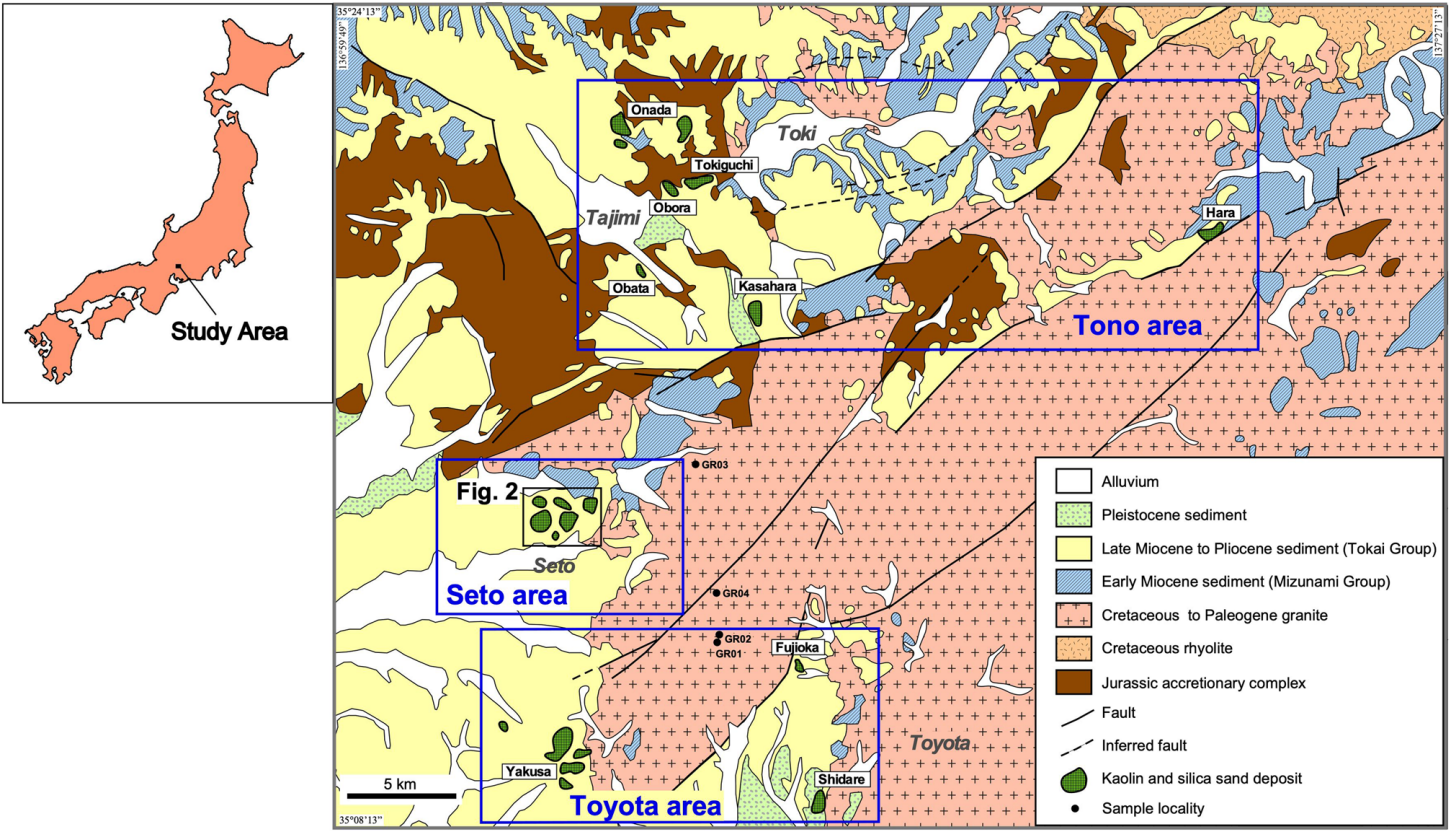
Geologic map of Seto-Tono district after Ref.^6^, distribution of kaolin and silica sand deposits and sample localities of basement rocks.

The cluster of sedimentary-hosted kaolin deposits distributed in the Seto-Tono district is the largest kaolin field in Japan. Although each sedimentary basin is smaller than 10 km^2^, 20 basins occur as a cluster in a 20 × 30 km area (Fig. 1). Kaolin mining in the Seto-Tono district dates back a 1000 years, with some mines still supplying kaolin as a raw material for porcelain and ceramics owing to its whiteness and highly viscous nature.

**Figure 2:**
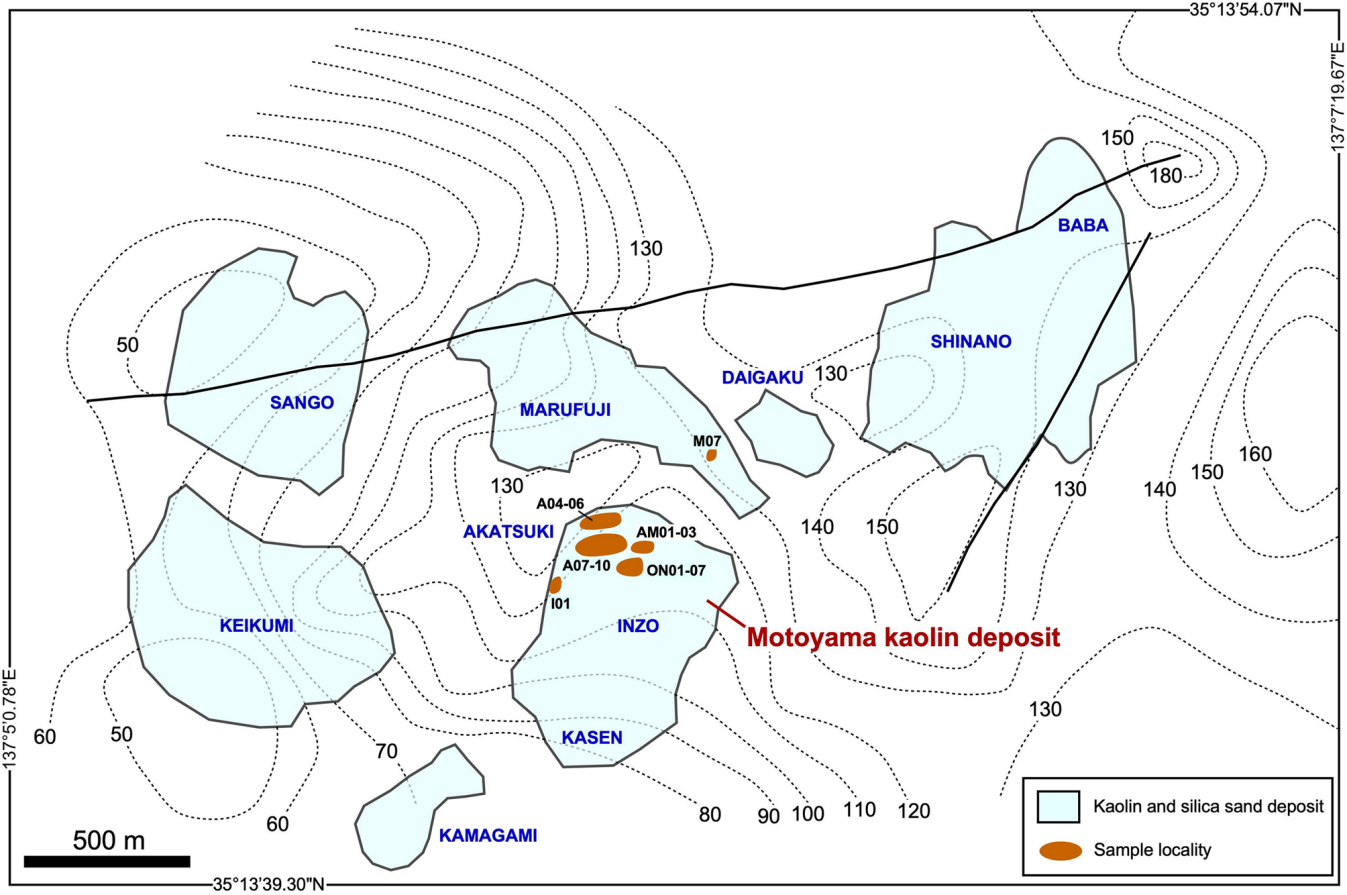
Distribution of kaolin and silica sand deposits in the Seto area and sample localities, based on aerial photographs of the Geospatial Information Authority of Japan and the personal communication with mining companies. The altitude (dotted lines, meter above the sea level) of basement granite beneath the Seto porcelain clay formation was drawn after Ref.^10^.

The basement of the Seto-Tono district consists of Jurassic accretionary complex and Cretaceous to Paleogene granite batholiths^6^. In the early Miocene, shallow marine sediments of Mizunami Group partly overlayed the basement and, in the late Miocene to Pliocene, non-marine sediments of Tokai Group widely overlayed the former geologic units^7,8^. The kaolin deposits are embedded in the Lower Tokai Group, Seto porcelain clay formation (PCF) and the equivalent Toki PCF. They were deposited 9–11 Ma, based on geochronology of tuff intercalated in the stratigraphy (Ref.^9^). During Pliocene, the Seto and Toki PCF were overlain by Yadagawa formation of the Upper Tokai Group, and the kaolin deposits survived erosion by the uplift after 3 Ma. This study focused on a Motoyama kaolin deposit which is exploited by Akatsuki, Inzo, and Kasen mines in the Seto area (Fig. 2).

### Sedimentary facies of the porcelain clay formations

Sedimentary facies of the Seto and Toki PCF have been extensively investigated by Refs.^10,11^, respectively. The studies indicate that the kaolin deposition occurred mainly in a lacustrine environment with subordinate fluvial incursions. A pedological study of the lacustrine sediments in the Tono area (Ref.^11^) revealed that the inland lakes used to be ephemeral bogs with alternating spells of stagnant water and completely dry conditions. The climate would have been tropical to subtropical during the deposition^11^.

The Seto PCF containing the Motoyama kaolin deposit was classified into the following nine lithologies (Ref.^10^); (1) poorly sorted matrix-supported gravel, (2) poorly sorted clast-supported gravel, (3) poorly sorted sand, (4) sand, medium to very coarse, (5) sandy gravel, (6) poorly sorted clay, (7) sand, mud, (8) well sorted clay, and (9) lignite. On the basis of their field occurrences, the following inferences regarding the depositional processes of the Seto PCF were made: in the early stage, sedimentary basins were separated into east and west parts by a mound of basement granite (see Fig. 2), and two alluvial fans comprising (4) to (6) were separately formed from northeast to southwest. Debris comprising (1) to (3) intermittently flowed into the alluvial fans. (6) was locally deposited in the periphery of the alluvial fans. In the later stage, the early sedimentary basins were reclaimed, and two alluvial fans merged into a large alluvial fan. (6)–(7) were widely deposited in the periphery of the alluvial fan and (8)–(9) were deposited in a bog beyond it along the flows from east to west. The PCF sedimented more slowly and gentle with time. In the latest stage, the inland lake expanded up to the western half of the Seto PCF distribution area.

### Motoyama kaolin deposit

**Figure 3:**
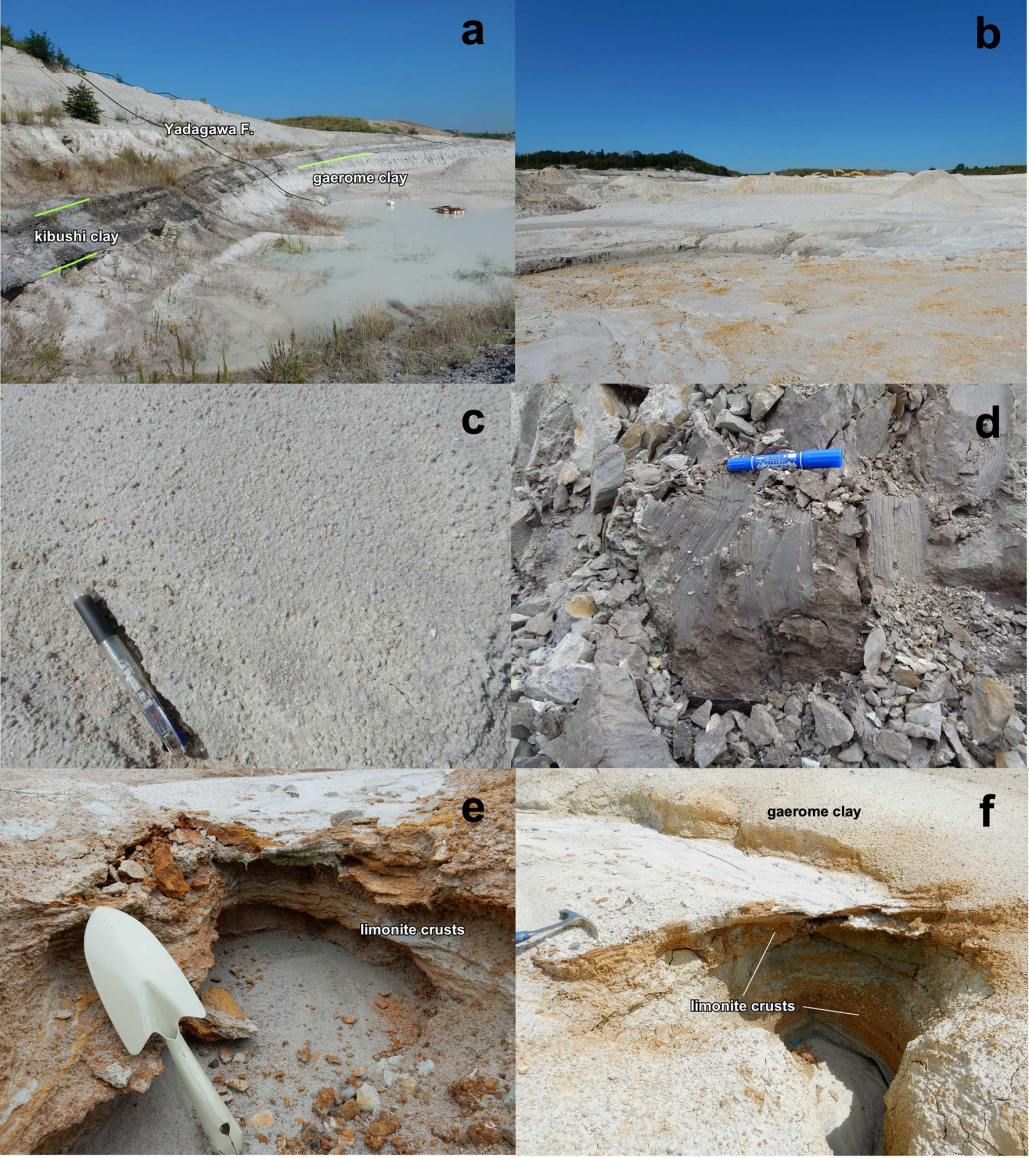
Field occurrences of Motoyama kaolin deposit. (**a**) An outcrop showing the stratigraphy of kibushi clay, gaerome clay, and Yadagawa formation (sand and gravel), (**b**) an open pit of the Akatsuki mine showing a shallow tray-shaped kaolin deposit, (**c**) gaerome clay, (**d**) kibushi clay, (**e**,**f**) limonite crusts.

**Figure 4:**
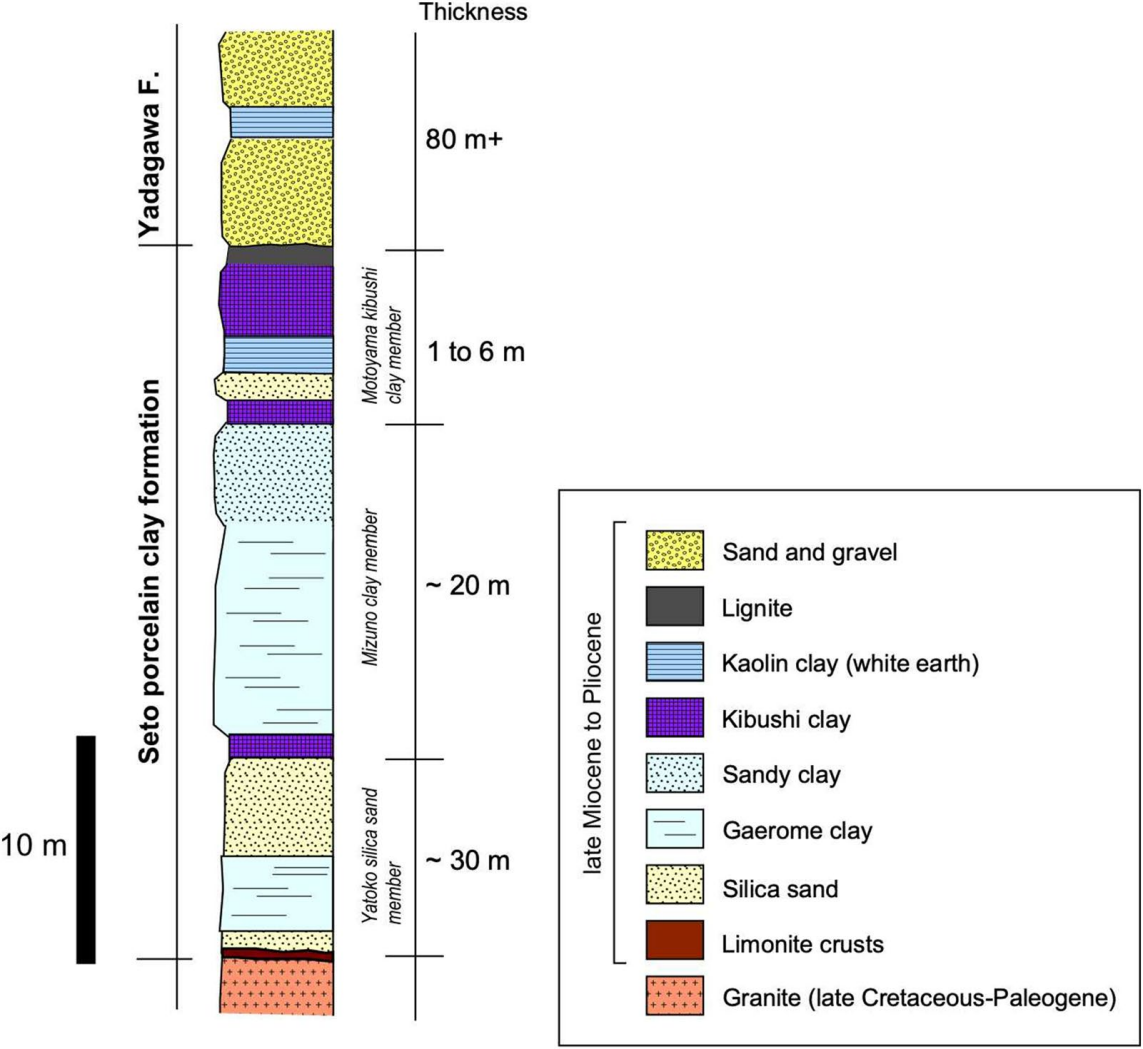
The integrated columnar section of Motoyama kaolin deposit has been modified based on Ref.^12^. Figure 3a is an outcrop ranging from the upper most Mizuno clay member to Yadagawa formation.

The Motoyama deposit is almost the same horizontally as that of the 500 m (EW) by 800 m (NS) oval open pit exploited by the mines. The Motoyama deposit is shaped like a shallow tray, and the strata is mostly flat (Fig. 3a,b). The stratigraphy is largely divided into three formations from the bottom to the top (Fig. 4): a late Cretaceous to Paleogene granite basement, the Seto PCF (15–45 m), and the Yadagawa formation (> 10 m) which is widely distributed in the Seto area. The outer margins of the Motoyama deposit are ambiguously bounded by gentle mounds of basement granite (Fig. 2); the field occurrence suggests that the deposit accumulated in a depression in the basement granite.

The Seto PCF comprises five kinds of sediments; kibushi clay, gaerome clay, kaolin clay (white earth), sandy clay, and silica sand. The gaerome clay is an unsorted and compact sediment consisting of coarse-grained quartz 1–5 mm in diameter (Fig. 3c) and fine-grained kaolin with small amounts of organic fragments, originating from arkose sandstone^10^. No carbonate minerals can be seen macroscopically. Gaerome literally means ‘frog’s eye’ in Japanese and is called so because the coarse-grained quartz resembles a frog's eye.

The gaerome clay constitutes the main part of the Seto PCF, having a total thickness of 5–15 m. It varies from light gray to white due to bleaching. The kibushi clay is a dark brown ligneous clayey sediment consisting of fine-grained kaolin and quartz with abundant carbonaceous materials. It is in the upper part of the formation, and is 1–6 m thick (Fig. 3a,d). Many in situ plant fossils are found in the gaerome clay, indicating a calm sedimentary environment. The peripheral part of the Motoyama deposit tends to be rich in quartz grains, whereas kaolin clay-rich parts (white earth) occur as lenticular layers and are irregularly distributed on a several meters-scale. The lower part of the Motoyama deposit comprises mainly silica sand, but their lateral continuity is often weak and lacking.

Limonite crusts 5–30 cm in thickness commonly appear along the unconformity plane between the gaerome clay beds and the basement granite (Figs. 3e,f, 4). The crusts are reddish-brown to orange massive aggregates consisting of pebbles cemented by goethite. The limonite crusts also occur as laminated thin beds or networks in gaerome clay beds in places, suggesting their concurrent or subsequent precipitation during the formation of the kaolin deposits. The weathered crusts of the basement granite underlying the kaolin deposit were generally subjected to kaolinization and converted to Fe-kaolinite bearing green saprolite. Incidentally, green saprolite (M07) collected from the neighboring Marufuji mine (Fig. 2) has been described elsewhere^13^.

## Results

The gaerome clay (A07) and kibushi clay (A08) specimens were elutriated with a 250-µm sieve to remove coarse crystals, pebbles and carbonaceous plant fossils. Finer fractions, which were not chemically treated, were examined and analyzed. The weight ratios of coarser fractions of A07 and A08 are 27% and 14%, respectively. The coarser fraction of A07 consists mostly of quartz up to 8 mm with subordinate feldspars, carbonaceous materials and chert pebbles, whereas A08 consists mostly of carbonaceous materials up to 15 mm, with subordinate quartz and feldspar grains up to 5 mm in diameter.

### X-ray diffraction analysis

**Figure 5:**
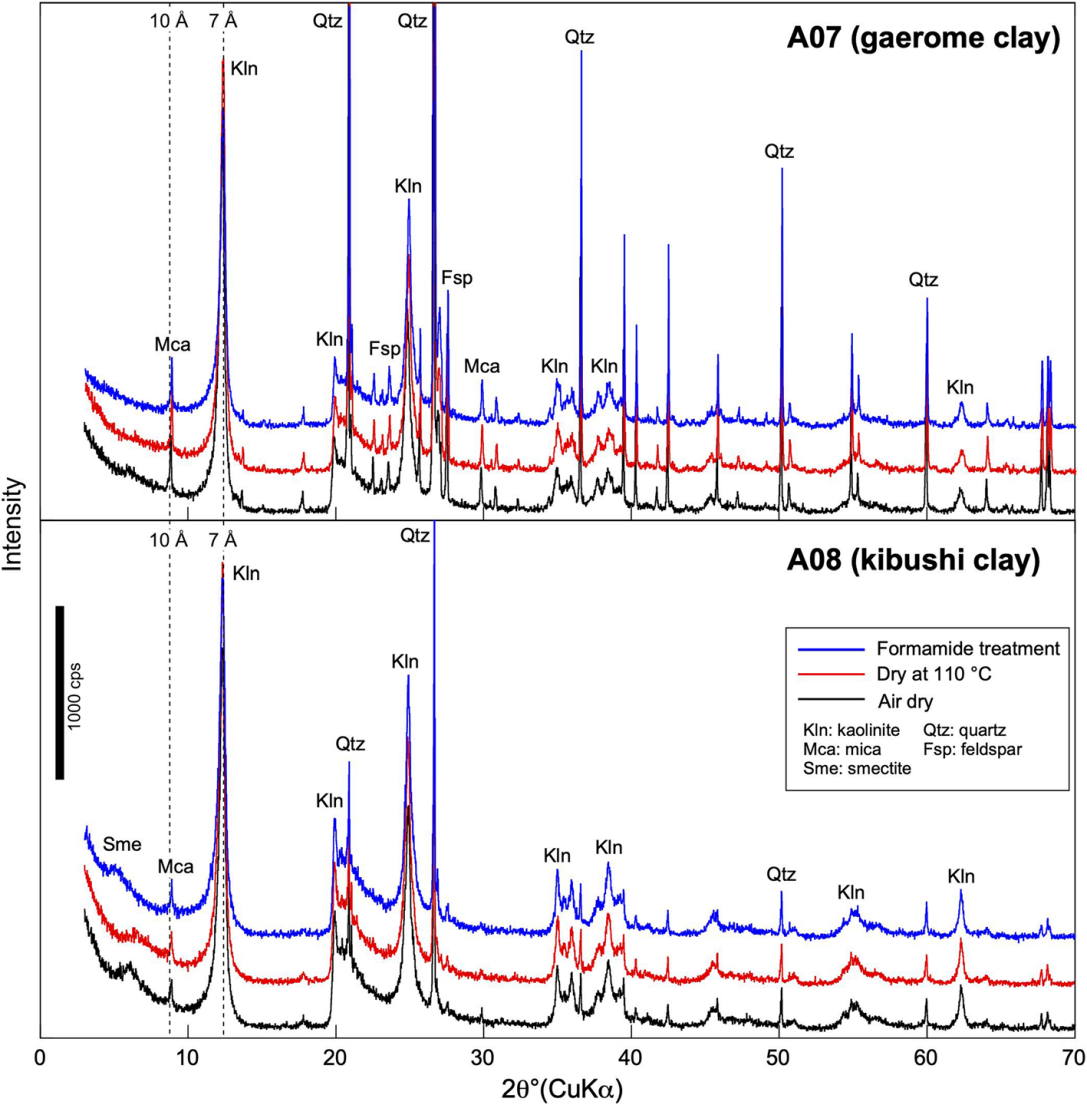
X-ray diffraction patterns of gaerome clay (A07) and kibushi clay (A08). The analyses were performed in the following conditions: dried in air, dried at 110 °C for 2 h, and treated with formamide.

The X-ray diffraction analysis (XRD) patterns showed that the gaerome and kibushi clays consist mostly of kaolinite, quartz with subordinate feldspars, mica, and minor smectite (Fig. 5).

On comparing the peaks of the kaolinite in formamide-treated samples with those of the samples dried in air or at 110 °C, the peak shift of the basal spacing from 7 to 10 Å which indicates the presence of halloysite^14^ could not be detected. Although the peaks from mica make the peak shift a little difficult to discern, it can be concluded that the gaerome (A07) and kibushi (A08) clays contain little halloysite.

### Scanning electron microscopy of kaolin clay ores

**Figure 6:**
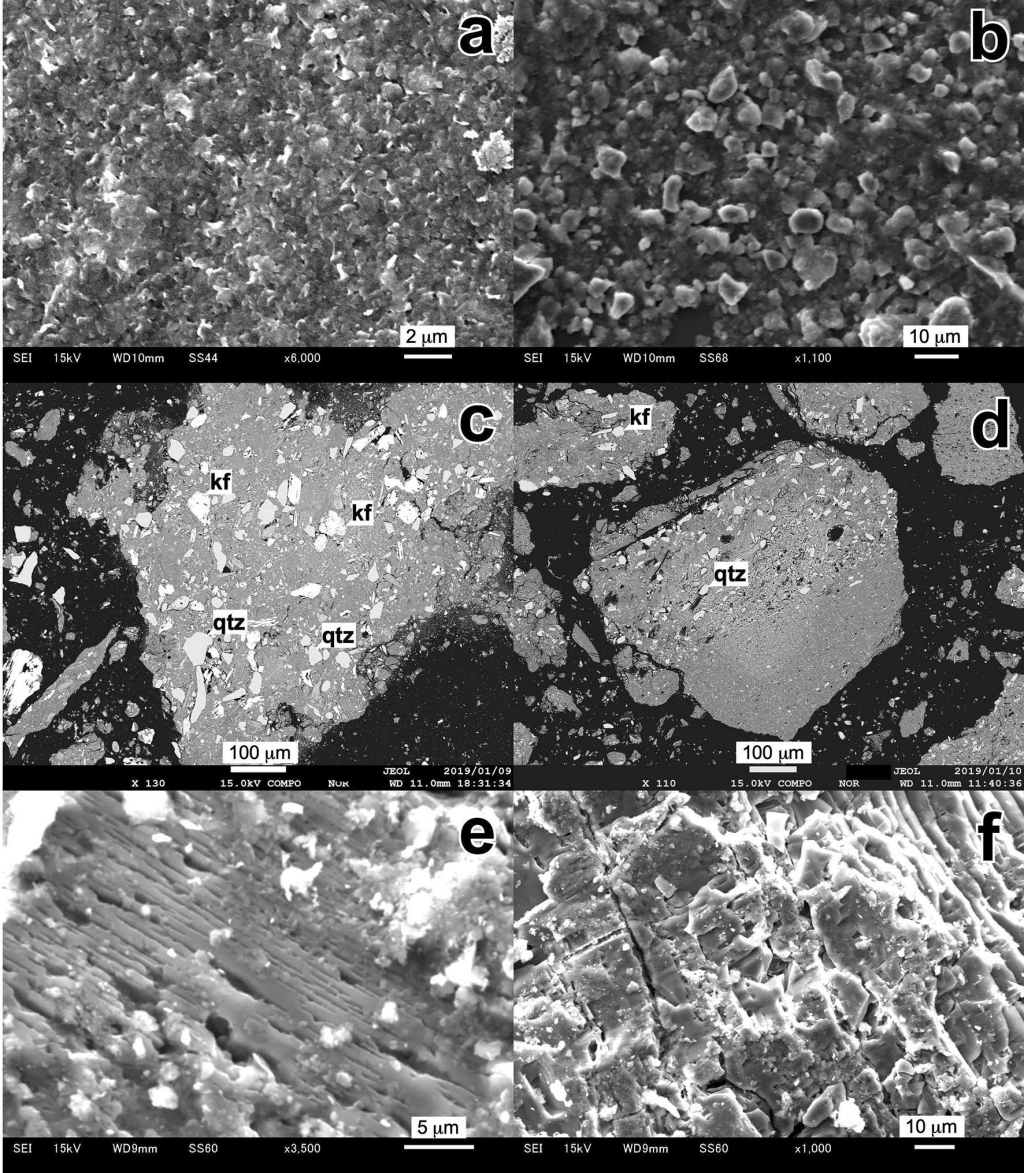
Electron micrographs (**a**,**b**) are secondary electron images (SEI) of gaerome and kibushi clay, respectively. Electron micrographs (**c**,**d**) are back scattered electron (BSE) images of polished sections of gaerome and kibushi clay, respectively. The aggregate grains comprise mainly fine-grained kaolinite (gray parts) and fragments of K-feldspar and quartz. Electron micrographs e and f are SEI of K-feldspar in gaerome clay. The decomposed surface of K-feldspar resembles an etched texture.

Kaolinite in the gaerome and kibushi clay occurs as irregularly shaped flakes ranging from < 1 to 10 µm (Fig. 6a,b). No thick booklets or hexagonal kaolinite are found in either clay. Quartz and K-feldspar grains ranging from 10 to 50 µm are common in the kibushi and even more common in the gaerome clay (Fig. 6c,d). Plagioclase is rare in both. K-feldspar often exhibits a rough surface as if it had been etched, as would be expected from acid degradation during kaolinization (Fig. 6e,f). Trace amounts of Fe–Ti oxides, anatase, zircon, monazite, xenotime, allanite, gypsum, and framboidal pyrite also occur in both types of clay.

### Field-emission electron microprobe analysis of kaolin clay

The kaolinite in gaerome and kibushi clays are fairly homogeneous in chemistry: the mean TiO_2_ wt% of the gaerome and kibushi clays are 0.51 (n = 25) and 0.70 (n = 44), respectively, while the mean Fe_2_O_3_(t) wt% are 1.04 and 1.05, respectively. The Fe_2_O_3_(t) range is similar to that in Georgian kaolin, whereas the TiO_2_ amounts are less than half of that in Georgian kaolin^15^. However, the Georgian kaolin measurements were performed on bulk ores, which contain between 0.6 and 3.3 wt% (mean 2.2 wt%) TiO_2_ derived from anatase^16^, rather than on its minerals.

### Occurrences of Fe–Ti oxides and Fe-hydroxide in kaolin clay ores

The gaerome and kibushi clays contain trace amounts of Fe–Ti oxides and Fe-hydroxide, which are likely to be decomposed residue of mafic silicates from the source sediments. To investigate redox conditions of kaolin clay ores, Fe–Ti oxides and Fe-hydroxide were separated from the ores (gaerome clay: A04, A05, A07 and kibushi clay: A06, A08) and investigated with a scanning electron microscope (SEM) and a field-emission electron microprobe analyzer (FE-EMPA).

**Figure 7:**
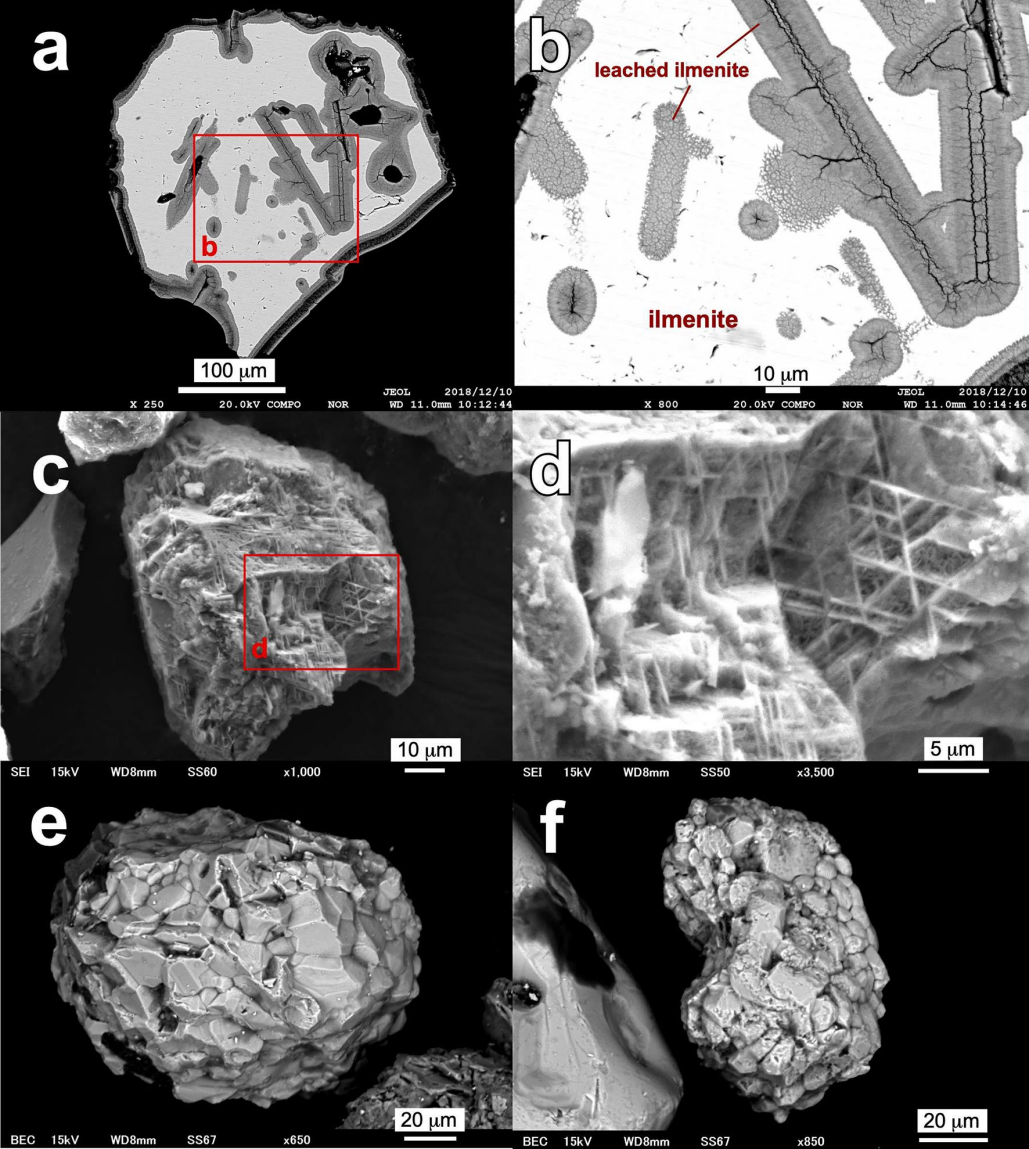
Electron micrographs showing the occurrences of ilmenite in gaerome and kibushi clay. BSE images (**a**,**b**) (a polished section) show leached ilmenite in an ilmenite grain of the gaerome clay (A07), indicating leached ilmenite as an alteration product of ilmenite. SEI (**c**,**d**) show a strongly corroded ilmenite grain in the kibushi clay (A08). BSE images (**e**,**f**) show framboid-like aggregates of ilmenite-pseudorutile in the kibushi clay (A08).

**Figure 8:**
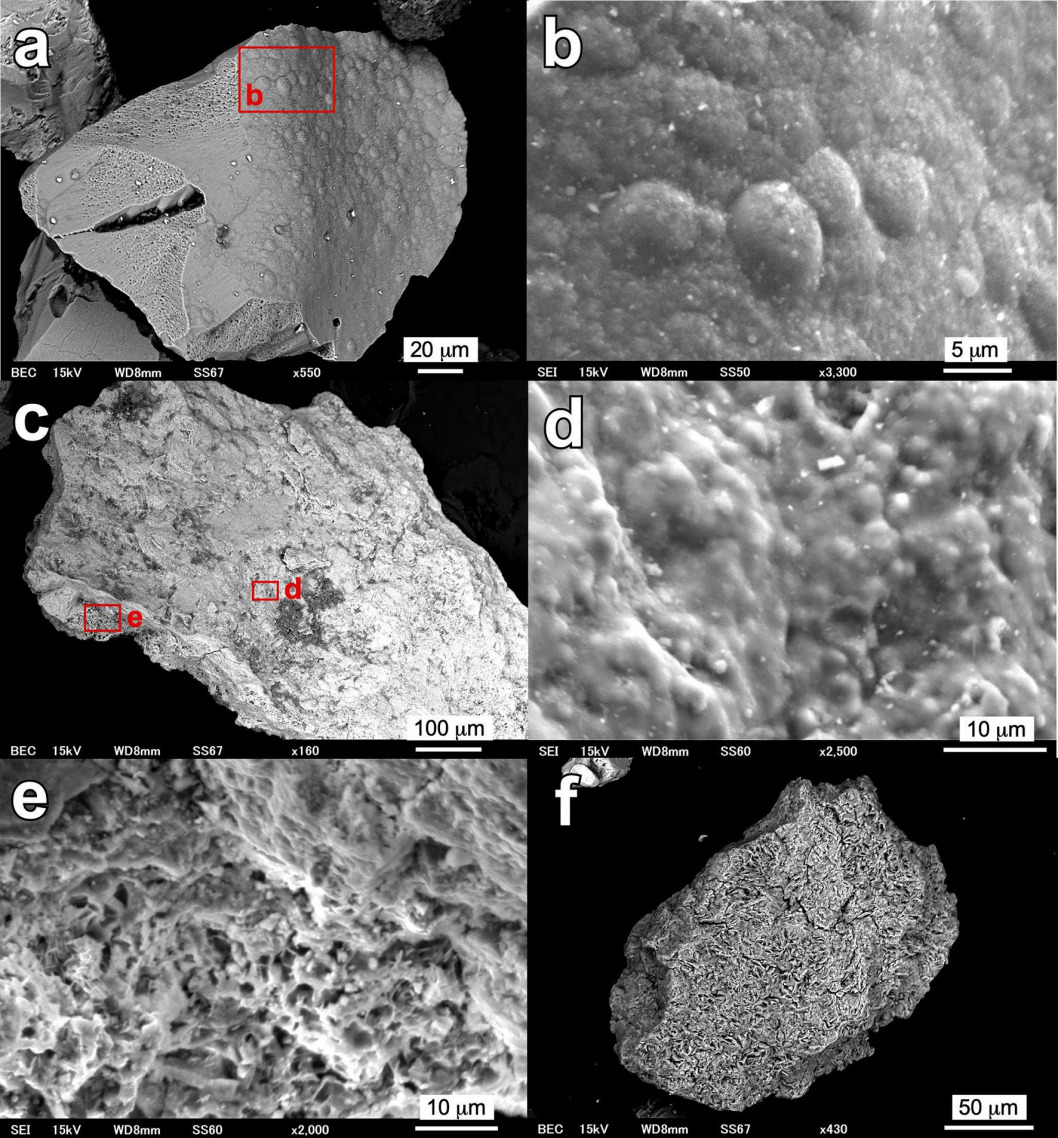
Electron micrographs showing the morphology of Fe-hydroxide in gaerome clay (A07). SEI (**a**–**d**) show rugged crusts and rounded lumps on the surface of Fe hydroxide. Electron micrographs (**e**) (SEI) and (**f**) (BSE image) show a sponge-like texture of the body of Fe-hydroxide grains.

The Fe–Ti oxides are mostly ilmenite or its alteration products. The ilmenite occurs as euhedral to subhedral grains ranging from several to several hundred µm in diameter. The ilmenite grains were often altered to leached ilmenite or pseudorutile along cracks (Fig. 7a,b), and some grains were strongly corroded, leaving a Ti–rich skeleton (Fig. 7c,d). Framboid-like aggregates of ilmenite-pseudorutile are also found in kibushi clay (Fig. 7e,f). Fe-hydroxide is found in only gaerome clay, but the mineral species could not be identified because distinct XRD peaks were not detected due to low crystallinity. The Fe-hydroxide occurs as irregular-shaped, porous grains ranging from several tens to a thousand µm in diameter. The surface of the grains is often coated by rugged crusts and has well-defined rounded lumps 2–3 µm in diameter (Fig. 8a–d). The body of the grains has a sponge-like texture (Fig. 8e,f).

### FE-EMPA analysis of Fe–Ti oxides

The chemical compositions of ilmenite and magnetite were recalculated as per (Ref.^17^). However, Fe–Ti oxides whose composition lay between ilmenite and pseudorutile or pseudorutile and rutile (anatase) could not be recalculated using that method. The former has been recognized as leached ilmenite and the latter as pseudorutile or leached pseudorutile^18^. Therefore, the compositions of leached ilmenite and pseudorutile (leached pseudorutile) were recalculated by assuming a molecular ratio of 9 oxygen to 3 titanium atoms.

**Figure 9:**
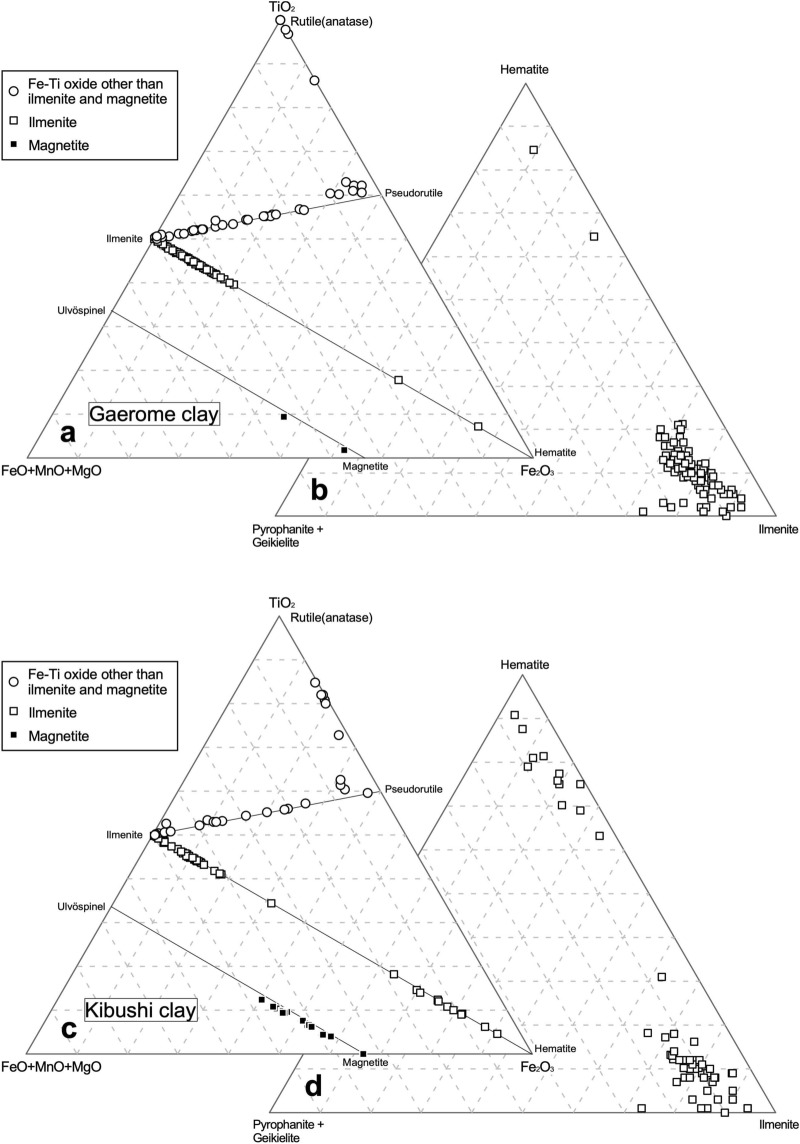
Chemical compositions of Fe–Ti oxides in gaerome and kibushi clay. Graphs (**a**,**c**): TiO_2_-(FeO + MnO + MgO)-Fe_2_O_3_ mol. ratios of Fe–Ti oxides. Graphs (**b**,**d**): ratios of hematite-(pyrophanite + geikielite)-ilmenite components of ilmenite. The ferric/ferrous ratios of the minerals were stoichiometrically calculated as described in the text.

The gaerome clay ores contain ilmenite, leached ilmenite, Fe-hydroxide, pseudorutile, leached pseudorutile, anatase and a trace amount of magnetite, whereas Fe-hydroxide and anatase could not be found in the kibushi clay. The recalculated compositions of Fe–Ti oxides and Ti oxide in the kibushi and gaerome clay ores are shown in Fig. 9. In Fig. 9a,c, most of the Fe–Ti oxides in the kibushi and gaerome clay ores fall on the tie lines connecting hematite-ilmenite, ilmenite-pseudorutile and magnetite-ulvöspinel, while the other Fe–Ti oxides lay between the ilmenite-pseudorutile line and rutile (anatase). On the basis of the mineral texture and compositional features of the Fe–Ti oxides, it can be assumed that successive oxidation and Fe-leaching probably occurred in the Motoyama deposits as follows^18^:

1 $$\begin{array}{*{20}l} {{\text{6Fe}}^{{{2} + }} {\text{TiO}}_{{3}} + {\text{ 3H}}_{{2}} {\text{O}} + { 1}.{\text{5O}}_{{2}} } \\ {{\text{ilmenite}}} \\ \end{array} \, \to \begin{array}{*{20}l} {{\text{2Fe}}^{{{3} + }}_{{2}} {\text{Ti}}_{{3}} {\text{O}}_{{9}} + {\text{ 2Fe}}\left( {{\text{OH}}} \right)_{{3}} } \\ {{\text{pseudorutile}}} \\ \end{array}$$

2 $$\begin{array}{*{20}l} {{\text{Fe}}^{{{3} + }}_{{2}} {\text{Ti}}_{{3}} {\text{O}}_{{9}} + {\text{ 3H}}_{{2}} {\text{O}}} \\ {{\text{pseudorutile}}} \\ \end{array} \to \begin{array}{*{20}l} {{\text{Fe}}^{{{3} + }} {\text{Ti}}_{{3}} {\text{O}}_{{6}} \left( {{\text{OH}}} \right)_{{3}} + {\text{ Fe}}\left( {{\text{OH}}} \right)_{{3}} } \\ {\text{leached pseudorutile}} \\ \end{array}$$

3 $$\begin{array}{*{20}l} {{\text{Fe}}^{{{3} + }} {\text{Ti}}_{{3}} {\text{O}}_{{6}} \left( {{\text{OH}}} \right)_{{3}} } \\ {\text{leached pseudorutile}} \\ \end{array} \to \begin{array}{*{20}l} {{\text{3TiO}}_{{2}} + {\text{ Fe}}\left( {{\text{OH}}} \right)_{{3}} } \\ {{\text{anatase}}} \\ \end{array}$$

In Fig. 9b,d, the hematite content in ilmenite varies widely from 0 to 89% in the kibushi clay ores and from 2 to 83% in the gaerome clay ores. It can be inferred that the Fe–Ti oxides are probably not detrital grains derived from the basement granite, because the granite is magnetite-free^19^ and our preliminary analysis showed the granitic ilmenite contain less than 1% hematite.

### Geochemistry of kaolin clay ores

To investigate the formation processes of the Motoyama deposit, gaerome, silicic gaerome (gaerome clay rich in quartz) and kibushi clay samples were assayed for C, H, N and S and isotopes of Fe, C and N.

**Figure 10:**
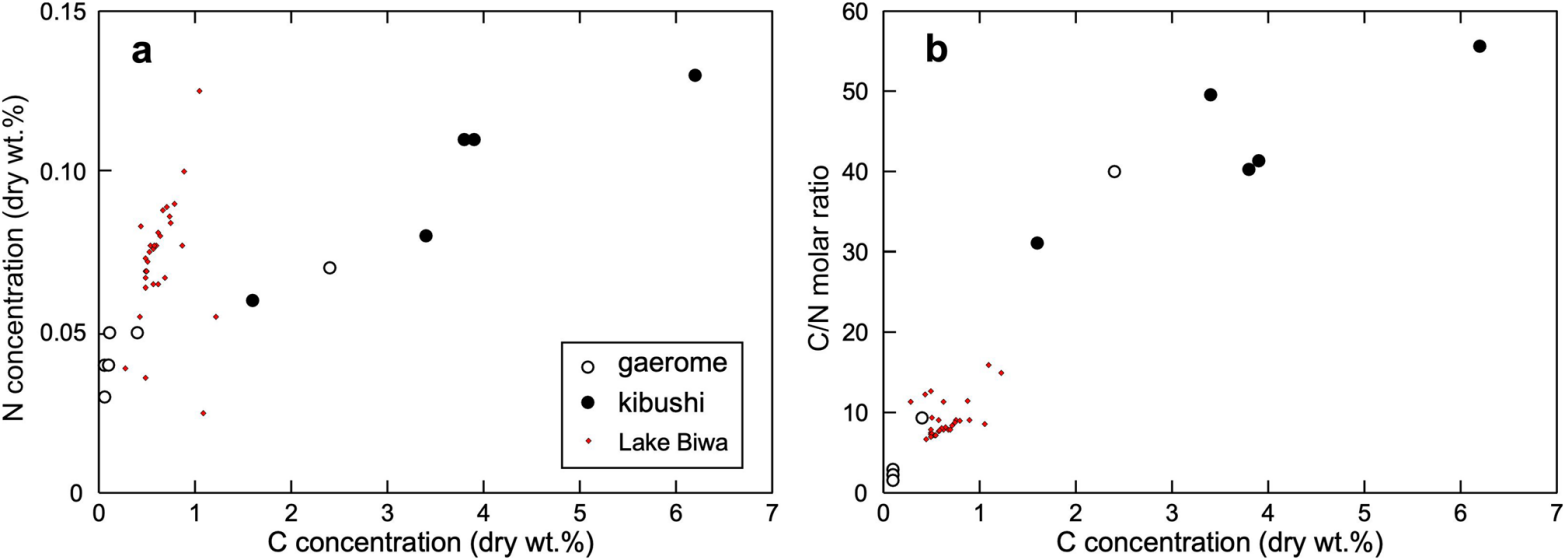
Graphs showing relationships between carbon and nitrogen in gaerome and kibushi clay. (**a**) C versus N (dry wt%), and (**b**) C (dry wt%) versus C/N molar ratio. The composition of Lake Biwa sediments^20^, which are modern lacustrine sediments 80 km west of the Seto area, is shown for comparison with kibushi and gaerome clay.

Five kibushi clay ores and six gaerome clay ores were quantitatively assayed for C, H, N and S. The results of the analyses are listed in Table 1. There is a positive correlation between C and N concentrations (Fig. 10a). Due to the paucity of carbonate carbon in the samples, the C and N are thought to be derived from organic matter. Correlations between H and N or H and C are not clear, because most of the hydrogen originated from the kaolin clay. The S concentration ranges from 0.002 to 0.08 wt%. The molar ratio of C/N ranges from 1.5 to 55.6 and has a positive correlation with the C concentration (Fig. 10b).

**Table 1:** Analytical results of C, TOC, H, N, S concentrations, and C, N, Fe stable isotopes.

No	A04	A05	A06	A07	A08	A09	A10	I01	AM01	AM02	AM03	
Type	grm	grm	kbs	grm	kbs	kbs	kbs	kbs	s-grm	s-grm	s-grm	
**(Dry %)**												
C	0.1	2.4	1.6	0.4	3.9	3.8	3.4	6.2	0.05	0.06	0.1	
TOC	0.1	2.3	1.5	0.4	3.7	3.4	3.3	6.0	< 0.1	< 0.1	0.1	
H	1.40	1.24	0.97	0.92	1.53	1.49	1.19	1.51	0.91	0.64	0.66	
N	0.04	0.07	0.06	0.05	0.11	0.11	0.08	0.13	0.04	0.03	0.05	
S	0.011	0.047	0.026	0.010	0.041	0.044	0.047	0.079	0.002	0.003	0.004	
C/N	2.9	40.0	31.1	9.3	41.4	40.3	49.6	55.6	1.5	2.3	2.3	
**(‰)**												
δ^13^C	− 26.4	− 27.9	− 27.5	− 26.5	− 27.5	− 27.3	− 27.4	− 28.4	− 25.8	− 26.3	− 25.2	
Error (±)	0.2	0.2	0.2	0.2	0.2	0.2	0.2	0.2	0.2	0.2	0.2	
δ^15^N	4.5	4.2	5.5	4.7	4.7	4.4	4.4	3.9	5.0	1.9	4.6	
Error ( ±)	0.5	0.2	0.2	0.2	0.2	0.2	0.2	0.2	0.3	0.3	0.3	
δ^56^Fe	0.112	0.231	0.288	0.143	0.192	–	–	–	–	–	–	
SD	0.076	0.080	0.052	0.018	0.060	–	–	–	–	–	–	

No	M07	ON01	ON02	ON03	ON04	ON05	ON06	ON07	GR01	GR02	GR03	GR04
Type	gsp	lmc	lmc	lmc	lmc	lmc	lmc	lmc	bgr	bgr	bgr	bgr
**(‰)**												
δ^56^Fe	0.259	0.184	− 0.372	0.905	− 0.325	− 0.530	0.352	− 0.102	0.261	− 0.074	0.082	0.256
SD	0.014	0.027	0.028	0.015	0.067	0.018	0.058	0.061	0.058	0.035	0.014	0.036

*TOC *total organic carbon, *grm *gaerome, *kbs *kibushi, *s-grm *silicic gaerome, *gsp *green saprolite, *lmc *limonite crust, *bgr *basement granite, *SD *standard deviation.

–: not analyzed, C/N: molar ratio of carbon and nitrogen.

**Figure 11:**
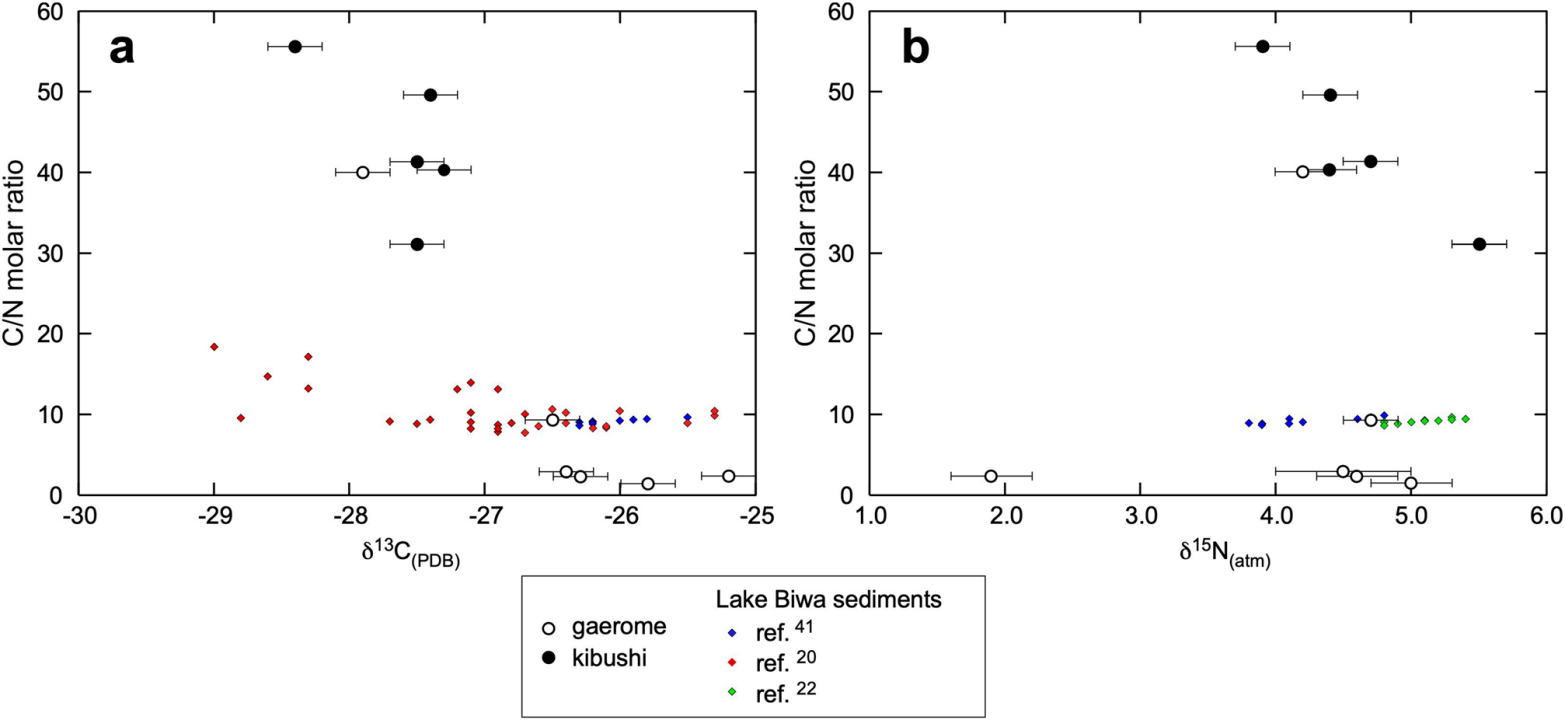
Graphs showing the relationships between δ^13^C and C/N molar ratios and between δ^15^N and C/N molar ratios in kibushi and gaerome clay and Lake Biwa sediments. In the compositions by Ref.^22^, data after 1950 were excluded to avoid anthropogenic effects such as nitrogen fertilizer.

The δ^13^C values of the kibushi and gaerome kaolin clay ores range from − 25.2 to − 28.4‰, and this range is common in C3 land plants and lacustrine algae^21^. Figure 11a shows a negative correlation between δ^13^C and C/N in the kibushi and gaerome clay ores.

The δ^15^N values of both clays range from 1.9 to 5.5‰, which is a common range for sediments^23^. The δ^15^N values are almost constant except for one value (1.9‰), and no systematic changes between δ^15^N and C/N are seen (Fig. 11b).

### Fe isotope compositions

The Fe isotope composition of the same five magnetic separates from the kibushi and gaerome clay ores described in the previous section were determined. In addition, the Fe isotope composition of a green saprolite, seven limonite crust and four fresh basement granite samples, as well as bulk samples, were determined.

**Figure 12:**
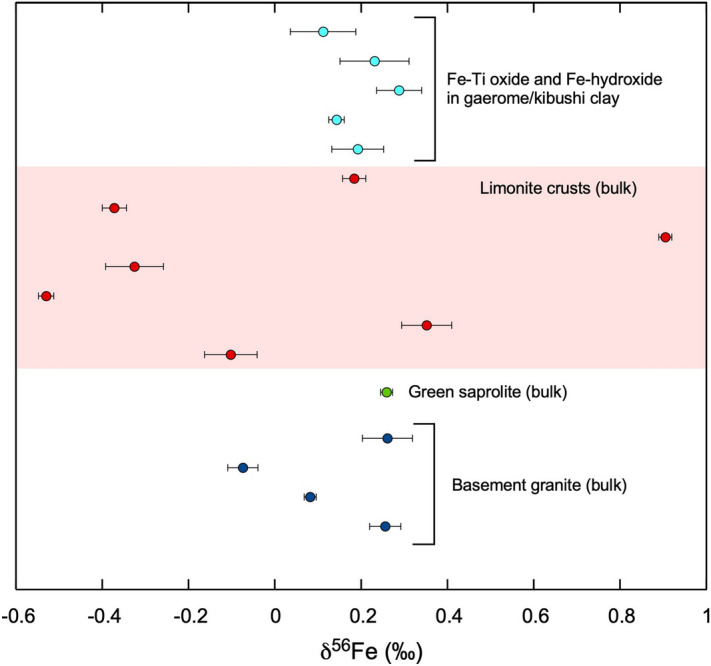
δ^56^Fe (‰) variations of constituents in Motoyama kaolin deposit and basement granites. The vertical axis describes the spatial relationship in Motoyama kaolin deposit. Pink indicates limonite crusts.

The δ^56^Fe values of Fe-bearing minerals in the kibushi and gaerome clay ores, the green saprolite and the basement granite are around 0.2 ‰ including analytical errors, except for one granite sample (− 0.07 ‰). In contrast, δ^56^Fe values in the limonite crust range from − 0.53 to 0.91 ‰, indicating significant isotope fractionation (Fig. 12).

## Discussion

On the basis of field occurrences, mineralogy and geochemical data, the most plausible origin of the Motoyama kaolin deposit was nitrification and acidification of lacustrine sediments due to microbial activity. Dilute nitric acid produced by the nitrification of ammonia could have caused decomposition of feldspars and micas in arkose sediments, resulting in Fe-leaching and intense kaolinization.

### Formation fields of kaolin clay

The unsorted nature of gaerome clays indicates that it was basically formed by post-depositional kaolinization rather than being external in origin; i.e., most kaolin clay was not derived from weathering products of basement rocks elsewhere, it was formed in situ. This is because gaerome clay readily separates into silica sand and kaolin clay when suspended in water. Thus, it is unlikely that the gaerome clay would have undergone transportation processes by streams. The transportation processes would occur mainly at the stage of arkose before kaolinization. Although, kibushi clay is well sorted, it would have been a stagnant bog sediment that experienced little transportation process, and it is reasonable to assume that kaolin clay was mostly formed in situ. The scarcity of halloysite in gaerome and kibushi clays also suggests that weathering products of basement rocks is not a main source of the kaolin clay. The formation of clay minerals commenced at the stage of arkose deposited in a lacustrine environment, which has a high permeability under almost neutral conditions, by illitization and smectitization of feldspars and micas. Due to the subsequent acidification of sediments, kaolinization progressed, and the permeability gradually decreased. However, since many quartz grains survived kaolinization, the sediments did not form an impermeable layer, and it is presumed that an acidic groundwater environment could be maintained.

### Fe oxidation and leaching in kaolin clay ores

The occurrences of Fe–Ti oxides (Fig. 7a–d) and their compositional trend (Fig. 9) in the gaerome and kibushi clay ores indicates that ilmenite crystallized first, and then Fe in ilmenite was oxidized and leached out.

As the gaerome and kibushi clays are largely depleted in Fe, presumably the limonite crusts are precipitates of the Fe that had leached out. Although, the temporal sequence of Fe oxidation/leaching and kaolinization is uncertain, their close field occurrence in the Motoyama deposit suggests that they happened concurrently. Therefore, elucidating the history of the iron is important in understanding the origin of the Motoyama deposit.

Atmospheric oxygen is unlikely to have been the oxidant because the lacustrine sediments would have been in suboxic or anoxic conditions due to the pore water having been mostly stagnant and isolated from the atmosphere. If the oxidant of the iron was hydrosulfuric acid, hydrothermal alteration would be the most probable origin, because hydrothermal solutions are the commonest source of sulfur and often induce kaolinization of rocks. In the case of hydrothermal kaolin deposits, clay minerals such as pyrophyllite and dickite that are often formed at higher temperatures and dissemination/veinlets of sulfides are common (for example Refs.^24,25^). In addition, a zoning of alteration degrees towards hydrothermal veins is occasionally observed in deposits (for example Ref.^26^). However, the evidence above has never been found in the Motoyama deposit. Another potential origin of kaolinization is low-temperature hydrothermal and pervasive alteration due to fluids of deep origin^27^. An example is the large-scale kaolin deposits in the Cornubia district of southwestern England^28^. If convective circulation of local groundwater occurred continuously in sedimentary basins of the Seto-Tono district driven by late-stage hydrothermal activities of the basement granite, the iron oxidation and kaolinization might have taken place. In that case, hydrothermal Fe–Si scale (iron hat) similar to limonite crusts might be formed on granites (for example Ref.^29^). However, the primary (higher temperature) hydrothermal alteration of the basement granites in the Seto-Tono district has never been reported as being similar to that of the Cornubia district. Moreover, the Seto-Tono district has been located on the trench side of the volcanic front, at least after Miocene^30^, and thus no igneous activity occurred there after Miocene. The tuff beds found in the kaolin deposits are presumably derived from volcanoes on the back-arc side. A possible hydrothermal source might be the fore-arc (Arima-type) hot spring of deep origin^31^, but no Arima-type hot spring is known to have existed in the district. Thus, no evidence of late Miocene hydrothermal activity has been found in the Seto-Tono district, and so iron oxidation by hydrosulfuric acid of hydrothermal origin is unlikely. As described below, the most plausible oxidant of iron was dilute nitric acid in groundwater, which may have been produced by microbial nitrification of plant-derived ammonia.

### Fe isotope fractionation

The speculation above is consistent with the significant Fe isotope fractionation found in limonite crusts; i.e., Fe isotope fractionation would occur in the processes of oxidation and leaching of iron, and it is likely that the activity of nitrate-dependent Fe oxidizing bacteria, whose activity has been well recognized in experiments (for example Refs.^32,33^), were involved. Furthermore, it is probable that ferric iron, which is barely soluble in neutral water, readily migrated as dissolved ferric nitrate in acidic conditions and precipitated along the boundary of pH and permeability (= an unconformity plane) as limonite crusts. Moreover, it is geologically improbable that the Fe isotope variation stemmed from the diversity of iron origins, because the lacustrine sediment would have virtually been a closed system with respect to Fe. As the Motoyama deposit was formed ten million years ago, it is unfeasible to obtain direct evidence of microbial activities (for example Ref.^34^), but the occurrence of framboid-like ilmenite-pseudorutile (Fig. 7e,f) and the regular texture of Fe-hydroxide (Fig. 8) imply the activity of Fe-oxidizing bacteria.

Numerous studies on Fe isotopes to date have shown that Fe isotope fractionation occurs in biotic or abiotic^35,36^. The studies on Fe isotope fractionation in soil have reported that Fe isotope compositions produced by the dissolution of silicates tended to be slightly lighter than the original isotope ratios^37,38^, whereas other studies on microbial Fe isotope fractionation with Fe oxidation have shown that Fe isotope compositions of Fe-hydroxide precipitates were generally heavier than that of ferrous iron in aqueous solution^39,40^. In the case of Motoyama deposits, it is difficult to identify at what stage Fe isotope fractionation occurred effectively from the present data, and more detailed examination is required.

### C and N concentrations and isotope compositions

To obtain clues to the origin of the Motoyama kaolin deposits from the C and N concentrations and isotope compositions, it is necessary to know how they have changed since then. Therefore, modern lacustrine sediments from Lake Biwa were compared as an analog of the original lacustrine sediments of the Seto area. Lake Biwa, which is located 80 km west of the Seto area, is larger and deeper (over 41.2 m deep) than any inland lake would have been in Miocene. A positive correlation between C and N contents in the Motoyama deposit means that these elements originally made up plant bodies. The fact that the C/N ratios of gaerome clay are significantly lower than those of kibushi clay is attributable to that the organic matter of gaerome clay was mainly derived from aquatic plants such as lacustrine algae, whereas that of kibushi clay was mainly land plants. The compositions of the Lake Biwa sediments are significantly less carbon-rich than those of the Motoyama deposit (Fig. 10). The C/N ratios are concentrated around 10^20,41^, suggesting that the organic matter is mostly of aquatic plants origin^42^.

In gaerome and kibushi clays, ^13^C was depleted with increasing C/N ratios, whereas ^13^C was depleted with depth in Lake Biwa sediments^20^. Such variations in carbon isotopes can be caused not only by changes in the source of carbon, but also the selective preservation of ^13^C-depleted organic compounds by bacterial and diagenetic degradation^43^. In the case of Lake Biwa sediments, it was concluded that the δ^13^C variation was mainly due to burial diagenesis^20^. However, in the case of Motoyama deposit, the above causes would be acting in combination.

Due to the narrow range of δ^15^N in the kaolin clay ores, little information was obtained regarding the origin of the Motoyama deposit. The C/N ratio of Lake Biwa sediments is almost constant throughout the δ^15^N range (Refs.^22,41^), because δ^15^N compositions are not sensitive for organic matter degradation^43^.

### Nitrification and acidification of lacustrine sediments

The degradation of feldspars and micas into kaolin clay is the main cause of the formation of the Motoyama kaolin deposit. Acidification (pH < 4.0) is necessary for the degradation of feldspars^44^. In case of hydrothermal argillic alteration, SO_2_ and HCl gases are the main cause of acidification^45^, whereas sediment (soil) acidification is generally caused by increased activity of acid anions such as H_2_CO_3_, HCO_3_^−^, SO_4_^2−^, NO_2_^−^ and NO_3_^−^. Acidification increases Al^3+^ activity in the system, resulting in kaolin clay formation. Of the acidic anions, NO_3_^−^ is the most likely agent for acidification of the lacustrine sediments.

HCO_3_^−^ is the major acid anion in soil solution, because H^+^ of H_2_CO_3_ is partially consumed by the reactions with bases in soils. Thus, the role of HCO_3_^−^ in acidification should be taken into account. The activity of HCO_3_^−^ in solution is a function of pH and CO_2_ partial pressure such that:

$$\left( {{\text{H}}^{ + } } \right)\left( {{\text{HCO}}_{{3}}^{ - } } \right) \, = {\text{ PCO}}_{{2}} \times {1}0^{{ - {7}.{81}}} ,$$

where the parentheses denote solution activity and PCO_2_ refers to atmospheric CO_2_ partial pressure (Ref.^46^). HCO_3_^−^ activity decreases with decrease in pH. Even greater than 3% CO_2_, HCO_3_^−^ activity is extremely low (ca. 5 μeq/L) at pH 4.0 (Refs.^46,47^). Therefore, HCO_3_^−^ would not be the major anion behind kaolinization, and would play only a secondary role through acidification. On the other hand, SO_4_^2−^ is a strong acid anion that would play a major role in the formation of hydrothermal kaolin deposits^48^. SO_4_^2−^, however, is not likely to cause kaolinization of the Motoyama deposit due to the above-mentioned geologic circumstances. Organic acids are important agents in mineral weathering near the surface^49,50^. Although, the behavior of organic acids in soil and sediments is not yet fully understood, organic acids released from plant roots play major roles in the decomposition of minerals and ingestion of nutrients from soil in the rhizosphere^51^. Therefore, it is reasonable to assume that they contributed a little to the formation of the Motoyama deposit.

### Formation processes of the Motoyama kaolin deposit

**Figure 13:**
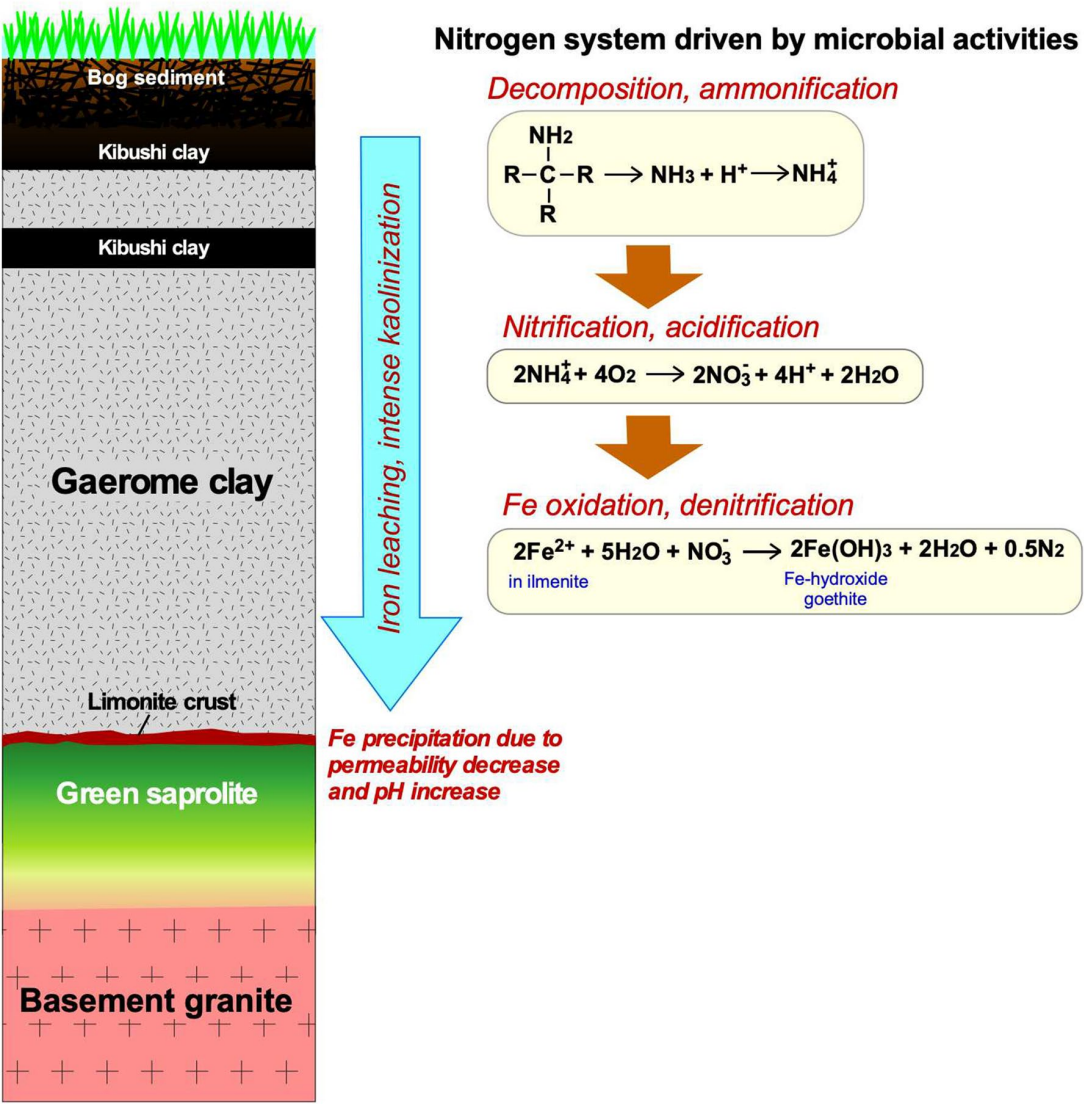
Schematic cross section of Motoyama kaolin deposits showing assumed material flow and chemical reactions.

A shallow inland lake was formed in the Miocene, and into it flowed weathering products derived mainly from the surrounding granite. The surface of the lake became covered with vegetation, resulting in it becoming like a bog with intermittent dry periods^11^. Anaerobic bacteria decomposed the vegetation, generating an abundance of ammonia. Microbial nitrification of the ammonia occurred in the subsurface under suboxic conditions, and NO_3_^−^ made the sediments acidic (pH < 4.0), resulting in kaolinization. Vegetation flourished whenever the lake refilled with water, but the above events kicked in during dry periods. Some NO_3_^−^ was used for oxidation of iron (denitrification) and, due to dilute nitric acid solutions, resultant ferric iron leached and migrated from the sediments into limonite crusts (Fig. 13).

The amount of nitrogen required for kaolinization under various assumptions can be estimated as follows:

$$\left( {{\text{N}}\,{\text{in}}\,{\text{plant}}} \right)/\left( {{\text{N}}\,{\text{in}}\,{\text{solution}}} \right) \times {\text{ E }} = {\text{ V,}}$$

where the parentheses denote weight %, E is the efficiency factor of the whole system, and V is the amount of nitric acid solution produced. The amount of nitrogen in solution depends on pH; the required amount of nitrogen increases with decreasing pH. Assuming that nitrogen in plants is 2 wt%, pH of solution = 3.0, temperature is 25 °C, and the efficiency factor is 0.3, 35 kg/m^2^ of plant material is necessary for the production of 15 m^3^ solution of dilute nitric acid, which is the column volume of the kaolin deposit. The values are reasonable given the biomass of wetlands (for example Ref.^52^), and kaolinization of lacustrine sediments can occur at least within decades.

The alternating spells of stagnant water and dry conditions in the Seto-Tono district were ideal for kaolinization by providing sufficient decomposition of bog plants. This special environment would have been made possible by the slow subsidence of the district that occurred in the late Miocene^53^, and the warmer climate at that time promoting the nitrification reactions^11^.

## Methods

### X-ray diffraction analysis

XRD was performed on gaerome and kibushi clays (Rigaku Smart Lab, Geological Survey of Japan: GSJ). The analytical protocol was as follows: CuKα radiation at 40 kV and 100 mA in a range 3–70° (2θ) with a scanning rate of 10° min^−1^, using a high-speed one-dimensional detector.

### Scanning electron microscopy

Kaolin clay powder, Fe–Ti oxides and Fe-hydroxide extracted from kaolin ores were observed with a scanning electron microscope (JEOL JSM-6610LV at GSJ). In addition, those were embedded in epoxy resin, one side was polished, and its surface was observed with a field-emission electron microprobe analyzer (JEOL JXA-8530F at GSJ).

The extraction of Fe–Ti oxides and Fe-hydroxide was performed using the following method: after elutriation of the ores with a 500-µm sieve, the finer fractions were added to distilled water in a beaker and the precipitates collected. Fe–Ti oxides were extracted from the precipitate with a Franz isodynamic separator. Ti oxide was also extracted from the precipitate by hand-picking.

### Field-emission electron microprobe analysis

Fe–Ti oxides and Fe-hydroxide extracted from kaolin ores embedded in the epoxy resin were quantitatively analyzed by FE-EMPA with a 15 kV accelerating voltage and a 20 nA beam current. The raw data were corrected using the ZAF method.

### C, H, N, and S concentration analysis

The analysis was conducted at Kyuden-Sangyo Co. Inc., Fukuoka, Japan. To analyze total organic carbon (TOC), samples were pretreated with 4 N HCl at 170 °C for two hours to remove carbonate. The C, H and N concentrations were analyzed by an elemental analyzer (CHN628, LECO). Sample (ca. 50 mg) was ground and placed in a tin capsule which was then burned in oxygen, oxidizing C to CO_2_, H to H_2_O, and N to NO_x_. The combustion gasses were collected in a ballast tank and homogenized. The amounts of H_2_O and CO_2_, and hence C and H, in the gas samples were determined by infrared spectroscopy. The NO_x_ was reduced to N_2_ by Cu and the amount of N_2_ determined by thermal conductivity. The S concentration was analyzed by ion chromatography (DIONEX ICS-1500, Thermo Scientific). A ground sample (ca. 5 mg) was placed in a crucible and pyrolyzed, converting S into SO_x_, using a pretreatment device (AQF-100, Mitsubishi Chemical). The combustion gas was absorbed into a solution of tartaric acid (10 ppm) and hydrogen peroxide (900 ppm), forming sulphate ions. The solution was injected into an ion chromatograph to quantify the SO_4_^2−^ and hence the S content.

### C and N isotope measurements

The samples used for the C, H, N and S analysis were also used for the C and N isotope measurements. For the C isotope measurements, an amount of sample containing 20–50 μg in carbon was placed in a tin capsule. The capsule was burned in the furnace (EA, Eurovector), and the emitted CO_2_ gas was separated in a separation column and introduced into a mass spectrometer (IsoPrime at the Kyuden-Sangyo Co., Inc.) with helium gas as the carrier. Each isotope ratio is the mean value of two measurements. The isotopic compositions are reported using the delta notation as the per-mil (‰) deviation of the ^13^C/^12^C ratio of the sample relative to that of PDB (Pee Dee Belemnite) using the following equation:

$$\delta^{{{13}}} {\text{C}}_{{{\text{PDB}}}} \left( \permil \right) \, = \, \left[ {\left( {^{{{13}}} {\text{C}}/^{{{12}}} {\text{C}}} \right)_{{{\text{sample}}}} /\left( {^{{{13}}} {\text{C}}/^{{{12}}} {\text{C}}} \right)_{{{\text{PDB}}}} - {1}} \right] \, \times { 1}000.$$

Isotopically calibrated acetanilide (δ^13^C = − 28.9 ‰) was used as a working standard.

For the N isotope measurement, an amount of sample containing 15 μg of carbon was placed in a tin capsule, and treated in the same way as the C isotope measurements. Each isotope ratio is the mean value of two measurements. The isotopic compositions are reported using the delta notation as the per-mil (‰) deviation of the ^15^N/^14^N ratio of the sample relative to that of the atmospheric N_2_ using the following equation:

$$\delta^{{{15}}} {\text{N}}_{{{\text{atm}}}} \left( \permil \right) \, = \, \left[ {\left( {^{{{15}}} {\text{N}}/^{{{14}}} {\text{N}}} \right)_{{{\text{sample}}}} /\left( {^{{{15}}} {\text{N}}/^{{{14}}} {\text{N}}} \right)_{{{\text{atm}}}} - {1}} \right] \, \times { 1}000.$$

Isotopically calibrated acetanilide (δ^15^N = − 0.9 ‰) was used as a working standard.

### Fe isotope measurements

The Fe isotope compositions were measured using a multi-collector ICP-MS (Neptune Plus, Thermo Scientific) at the Research Institute for Humanity and Nature, Kyoto, Japan. Instrumental mass bias was corrected using the standard-sample-standard bracketing method^54^. The analyses were performed in middle-resolution mode (*M*/Δ*M* = 8000–9000) in order to eliminate the major isobar interferences of ^40^Ar^14^N^+^, ^40^Ar^16^O^+^ and ^40^Ar^16^OH^+^ on ^54^Fe, ^56^Fe and ^57^Fe, respectively^54^. The interferences of ^54^Cr^+^ on ^54^Fe^+^ and ^58^Ni^+^ on ^58^Fe^+^ were monitored using ^52^Cr^+^ and ^60^Ni^+^, and the contributions were corrected using the isotopic abundance ratios of ^54^Cr/^52^Cr = 0.0282 and ^58^Ni/^60^Ni = 2.616 (Ref.^55^). The isotopic compositions are reported using the delta notation as the per-mil (‰) deviation of the ^56^Fe/^54^Fe ratio of the sample relative to that of IRMM-014b (Institute for Reference Materials and Measurements) using the following equation:

$$\delta^{{{56}}} {\text{Fe}}_{{{\text{IRMM}} - 0{\text{14b}}}} \left( \permil \right) \, = \, \left[ {\left( {^{{{56}}} {\text{Fe}}/^{{{54}}} {\text{Fe}}} \right)_{{{\text{sample}}}} /\left( {^{{{56}}} {\text{Fe}}/^{{{54}}} {\text{Fe}}} \right)_{{{\text{IRMM}} - 0{\text{14b}}}} - {1}} \right] \, \times { 1}000.$$

The reproducibility of the standard during these analyses was ± 0.10 ‰ (2σ).

## References

[1] Schroeder PA, Erickson G (2014). Kaolin: From ancient porcelains to nanocomposites. Elements.

[2] Prasad MS, Reid KJ, Murray HH (1991). Kaolin: Processing, properties and applications. Appl. Clay Sci..

[3] Dill HG (2016). Kaolin: Soil, rock and ore from the mineral to the magmatic, sedimentary and metamorphic environments. Earth Sci. Rev..

[4] Hurst VJ, Pickering SM (1997). Origin and classification of coastal plain kaolins, southeastern USA, and the role of groundwater and microbial action. Clays Clay Mineral..

[5] Shelobolina ES, Pickering SM, Lovley DR (2005). Fe-cycle bacteria from industrial clays mined in Georgia USA. Clays Clay Mineral..

[6] Geological Survey of Japan, AIST (ed.). *Seamless digital geological map of Japan, April 6, 2020 version.* https://gbank.gsj.jp/geonavi/geonavi.php#12,35.27798,137.18986 (2020).

[7] Makiyama, J. *Regional Geology of Japan* Chubu Region **4**, (Asakura Publishing, 1950).

[8] Nakayama K (1985). Sedimentary basins of the Tokai Group in the southern part of Toki City, Gifu Prefecture, Central Japan. Assoc. Geol. Collab. Jpn..

[9] Yoshida, S., Nakayama, K. & Danhara, T. Fission-track ages of the lower part of the Seto Group, Aichi and Gifu Prefectures, central Japan. *Jpn. Earth. Planet. Sci. Jt. Meet. Abstr.* 584 (1997).

[10] Nakayama K (1991). Depositional process of the Neogene Seto Porcelain Clay Formation in the northern part of Seto City, Central Japan. J. Geol. Soc. Jpn..

[11] Hatano N, Yoshida K (2017). Sedimentary environment and paleosols of middle Miocene fluvial and lacustrine sediments in central Japan: Implications for paleoclimate interpretations. Sediment. Geol..

[12] Otsuka, T., Kondo, Y., Sasaki, M., Takada, Y. & Shimosaka, Y. In *Silica Sand and Refractory Clay Deposits in Seto City and the Vicinity.* 1–50 (Aichi Prefecture & Geological Survey of Japan, 1968).

[13] Jige M, Takagi T, Takahashi Y, Kurisu M, Tsunazawa Y, Morimoto K, Hoshino M, Tsukimura K (2018). Fe-kaolinite in granite saprolite beneath sedimentary kaolin deposits: A mode of Fe substitution for Al in kaolinite. Am. Mineral..

[14] Churchman GJ, Whitton JS, Claridge GGC, Theng BKG (1984). Intercalation method using formamide for differentiating halloysite from kaolinite. Clays Clay Mineral..

[15] Kesler TL (1956). Environment and origin of the Cretaceous kaolin deposits of Georgia and South Carolina. Econ. Geol..

[16] Schroeder PA, Shiflet J (2000). Ti-bearing phases in the Huber formation, an East Georgia kaolin deposit. Clays Clay Mineral..

[17] Stormer JC (1983). The effects of recalculation on estimates of temperature and oxygen fugacity from analyses of multicomponent iron-titanium oxides. Am. Mineral..

[18] Mücke A, Bahadra Chaudhuri JN (1991). The continuous alteration of ilmenite through pseudorutile to leucoxene. Ore Geol. Rev..

[19] Ishihara S, Wu C (2001). Genesis of late Cretaceous-Paleogene granitoids with contrasting chemical trends in the Chubu District, Central Japan. Bull. Geol. Surv. Jpn..

[20] Ishiwatari R, Uzaki M (1987). Diagenetic changes of lignin compounds in a more than 0.6 million-years-old lacustrine sediment (Lake Biwa, Japan). Geochim. Cosmochim. Acta..

[21] Meyers PA, Lallier-Vergès E (1999). Lacustrine sedimentary organic matter records of Late Quaternary paleoclimates. J. Paleolimnol..

[22] Ogawa NO, Koitabashi T, Oda H, Nakamura T (2001). Fluctuation of nitrogen isotope ratio of gobiid fish (Isaza) specimens and sediments in Lake Biwa, Japan, during the 20th century. Limnol. Oceanogr..

[23] Hoefs, J. *Stable Isotope Geochemistry*, 8th edn., 66–72 (Springer, New York, 2018).

[24] Abedini A, Calagari AA (2016). Geochemical characteristics of the Arabshah kaolin deposit, Takab geothermal field, NW Iran. Arab. J. Geosci..

[25] Fujii N, Tsukimura K, Julio JM (1989). Mode of occurrence and genetic processes of the Iriki kaolin deposit, southern Kyushu. Bull. Geol. Surv. Jpn..

[26] Takagi T, Koh S-M, Kim M-Y, Naito K, Sudo S (2000). Geology and hydrothermal alteration of the Milyang pyrophyllite deposit, southeast Korea. Resour. Geol..

[27] Robb, L. *Introduction to ore-forming processes*, 2nd edn., 260–262 (Wiley Blackwell, Hoboken, 2020).

[28] Alderton, D.H.M. Mineralization associated with the Cornubian granite batholith. In *Mineralization in the British Isles* (eds. Pattrick, R.A.D. & Polya, D.A.), 270–354 (Chapman & Hall, London, 1993).

[29] Manceau A, Ildefonse P, Hazemann J-L, Flank A-M, Gallup D (1995). Crystal chemistry of hydrous iron silicate scale deposits at the Salton Sea geothermal field. Clays Clay Mineral..

[30] Miura D, Wada Y (2007). Middle Miocene ash-flow calderas at the compressive margin of southwest Japan arc: Review and synthesis. J. Geol. Soc. Jpn..

[31] Kusuda C, Iwamori H, Nakamura H, Kazahaya K, Morikawa N (2014). Arima hot spring waters as a deep-seated brine from subducting slab. Earth Planets Space.

[32] Straub KL, Benz M, Schink B, Widdel F (1996). Anaerobic, nitrate-dependent microbial oxidation of ferrous iron. Appl. Environ. Microbiol..

[33] Weber KA, Picardal FW, Roden EE (2001). Microbially catalyzed nitrate-dependent oxidation of biogenic solid-phase Fe(II) compounds. Environ. Sci. Technol..

[34] Kappler A, Straub KL (2005). Geomicrobiological cycling of iron. Rev. Mineral. Geochem..

[35] Johnson CM, Beard BL (2006). Fe isotopes: An emerging technique for understanding modern and ancient biogeochemical cycles. GSA Today.

[36] Brantley SL, Liermann LJ, Guynn RL, Anbar A, Icopini GA, Barling J (2004). Fe isotopic fractionation during mineral dissolution with and without bacteria. Geochim. Cosmochim. Acta..

[37] Brantley SL, Liermann L, Bullen TD (2001). Fractionation of Fe isotopes by soil microbes and organic acids. Geology.

[38] Emmanuel S, Erel Y, Matthews A, Teutsch N (2005). A preliminary mixing model for Fe isotopes in soils. Chem. Geol..

[39] Croal JR, Johnson CM, Beard BL, Newman DK (2004). Iron isotope fractionation by Fe(II)-oxidizing photoautotrophic bacteria. Geochim. Cosmochim. Acta..

[40] Swanner ED, Bayer T, Wu W, Hao L, Obst M, Sundman A, Byrne JM, Michel FM, Kleinhanns IC, Kappler A, Schoenberg R (2017). Iron isotope fractionation during Fe(II) oxidation mediated by oxygen-producing marine cyanobacterium *Synechococcus* PCC 7002. Environ. Sci. Technol..

[41] Hyodo F (2008). Changes in stable isotopes, lignin-derived phenols, and fossil pigments in sediments of Lake Biwa, Japan: Implications for anthropogenic effects over the last 100 year. Sci. Total Environ..

[42] Meyers PA (1994). Preservation of elemental and isotopic source identification of sedimentary organic matter. Chem. Geol..

[43] Lehmann MF, Bernasconi SM, Barbieri A, McKenzie JA (2002). Preservation of organic matter and alteration of its carbon and nitrogen isotope composition during simulated and in situ early sedimentary diagenesis. Geochim. Cosmochim. Acta..

[44] Blum AE, Stillings LL (1995). Feldspar dissolution kinetics. Rev. Mineral..

[45] Reed, M.H. Hydrothermal alteration and its relationship to ore fluid composition. In *Geochemistry of Hydrothermal Ore Deposits* 3rd edn (ed. Barnes, H. L.), 303–365 (Wiley, Hoboken, 1997).

[46] Reuss, J. O. & Johnson, D. W. *Acid Deposition and the Acidification of Soils and Waters**Ecological Studies*, vol. 59, 1–30 (Springer, New York, 1986).

[47] Wilkin RT, Digiulio DC (2010). Geochemical impacts to groundwater from geologic carbon sequestration: Controls on pH and inorganic carbon concentrations from reaction path and kinetic modeling. Environ. Sci. Technol..

[48] Nagasawa, K. In *Clays and Clay Minerals of Japan**Development in Sedimentology*, vol. 26 (eds. Sudo, T. & Shimoda, S.), 189–219 (Elsevier, Amsterdam, 1978).

[49] Bennett PC, Melcer ME, Siegel DI, Hassett JP (1988). The dissolution of quartz aqueous solutions of organic acids at 25°C. Geochim. Cosmochim. Acta..

[50] Welch SA, Ullman WJ (1996). Feldspar dissolution in acidic and organic solutions: Compositional and pH dependence of dissolution rate. Geochim. Cosmochim. Acta..

[51] Jones DL (1998). Organic acids in the rhizosphere—a critical review. Plant Soil.

[52] Whigham DF, Simpson RL (1992). Annual variation in biomass and production of a tidal freshwater wetland and comparison with other wetland systems. Virginia J. Sci..

[53] Nakayama K (1996). Depositional processes for fluvial sediments in an intra-arc basin: An example from the Upper Cenozoic Tokai Group in Japan. Sediment. Geol..

[54] Weyer S, Schwieters JB (2003). High precision Fe isotope measurements with high mass resolution MV-ICPMS. Int. J. Mass Spectrom..

[55] Beard BL, Johnson CM (1999). High precision iron isotope measurements of terrestrial and lunar materials. Geochim. Cosmochim. Acta..

